# Fibroblast growth factor‐23 and parathyroid hormone suppress small intestinal magnesium absorption

**DOI:** 10.14814/phy2.15247

**Published:** 2022-04-06

**Authors:** Nasisorn Suksridechacin, Narongrit Thongon

**Affiliations:** ^1^ Division of Physiology Department of Biomedical Sciences Faculty of Allied Health Sciences Burapha University Chonburi Thailand; ^2^ Biodiversity Research Centre Thailand Institute of Scientific and Technological Research Pathumthani Thailand

**Keywords:** hormonal regulation, magnesium absorption, parathyroid hormone, fibroblast growth factor‐23, magnesium supplement

## Abstract

In the present study, we examined the systemic and direct effects of parathyroid hormone (PTH) and fibroblast growth factor‐23 (FGF‐23) on duodenal, jejunal, and ileal Mg^2+^ absorption. The rats were injected with FGF‐23 or PTH for 5 h before collecting the duodenum, jejunum, and ileum for Mg^2+^ transport analysis in Ussing chambers. The duodenum, jejunum, and ileum were directly exposed to FGF‐23, PTH, or FGF‐23 plus PTH with or without cell signaling inhibitors for 150 min in Ussing chambers prior to performing the Mg^2+^ transport study. The small intestinal tissues were also subjected to western blot analyses for FGF receptor (FGFR), PTH receptor (PTHR), Klotho, transient receptor potential melastatin 6 (TRPM6), and cyclin as well as the cystathionine β‐synthase domain divalent metal cation transport mediator 4 (CNNM4) expression. The small intestine abundantly expressed FGFR and PTHR proteins, whereas, Klotho was not expressed in rat small intestine. Systemic PTH or FGF‐23 injection significantly suppressed transcellular Mg^2+^ transport in the duodenum and jejunum. Direct FGF‐23‐, PTH‐, or FGF‐23 plus PTH exposure also suppressed transcellular Mg^2+^ absorption in the duodenum and jejunum. There was no additional inhibitory effect of PTH and FGF‐23 on intestinal Mg^2+^ absorption. The inhibitory effect of PTH, FGF‐23, or FGF‐23 plus PTH was abolished by Gö 6850. Systemic PTH‐ or FGF‐23‐injection significantly decreased membranous TRPM6 expression, but increased cytosolic CNNM4 expression in the duodenum, jejunum, and ileum. In the present study, we propose a novel magnesiotropic action of PTH and FGF‐23 by modulating small intestinal Mg^2+^ absorption.

## Graphical abstract

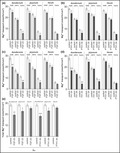

## 1 INTRODUCTION

Mg^2+^ plays an important role in more than 800 essential enzymatic activities in the human body (de Baaj et al., 2015). Dietary intake is the sole source of Mg^2+^; therefore, bulk absorption of Mg^2+^ in the small intestine is vital for health. Previous studies revealed that the duodenum, jejunum, and ileum absorb Mg^2+^ through both transcellular and paracellular mechanisms (Suksridechacin et al., 2020; Thongon et al., 2016). Transcellular Mg^2+^ transport proceeds through apical Mg^2+^ uptake by transient receptor potential melastatin 6 (TRPM6) and basolateral Mg^2+^ extrusion by cyclin and cystathionine β‐synthase domain divalent metal cation transport mediator 4 (CNNM4) (de Baaj et al., 2015). TRPM6 and CNNM4 were markedly expressed in the murine (Suksridechacin et al., 2020; Voets et al., 2004) and human (Schlingmann et al., 2002) small intestine. Currently, the regulatory mechanism of small intestinal Mg^2+^ absorption is poorly understood.

The calcemic and phosphatemic effects of parathyroid hormone (PTH) and fibroblast growth factor‐23 (FGF‐23) were previously described. In hypocalcemia, high circulating PTH triggers the osteoclast nuclear factor‐κB pathway to stimulate bone resorption, thereby releasing Ca^2+^, P_i_, and Mg^2+^ into the circulation (Leaf & Christov, 2019; Zofkova & Kancheva, 1995). PTH stimulates 1,25‐dihydroxy vitamin D_3_ [1,25(OH)_2_D_3_] production, which subsequently induces small intestinal Ca^2+^ absorption (Fleet et al., 1994). PTH further activates renal Ca^2+^ and Mg^2+^ reabsorption (Leaf & Christov, 2019; Vetter & Lohse, 2002). Simultaneously, PTH and P_i_ trigger osteocyte‐derived FGF‐23 production. As a negative feedback regulator, FGF‐23 abolishes 1,25(OH)_2_D_3_‐induced intestinal Ca^2+^ absorption (Khuituan et al., 2012) to prevent hypercalcemia. In renal tubules, PTH and FGF‐23 down‐regulate the Na^2+^‐dependent P_i_ cotransporters, (NaPi)‐IIa and NaPi‐IIc, and increase urinary P_i_ excretion (Leaf & Christov, 2019) to prevent hyperphosphatemia. However, these endocrine functions in the bone–intestine–kidney axis probably induce hypermagnesemia by means of bone resorption and renal reabsorption. Nevertheless, the effect of PTH and FGF‐23 on intestinal Mg^2+^ absorption is unknown. We hypothesize that PTH and/or FGF‐23 may suppress small intestinal Mg^2+^ absorption in order to prevent hypermagnesemia.

The effects of PTH on intestinal Mg^2+^ absorption are currently controversial. Chronic PTH injection significantly increased intestinal Mg^2+^ absorption in rats (Fleet et al., 1994) and humans (Hulter & Peterson, 1984). Meanwhile, acute PTH exposure may suppress intestinal Mg^2+^ absorption through direct activation of mucosal HCO_3_^¯^ secretion (Laohapitakworn et al., 2011), a reported inhibitory factor for small intestinal Mg^2+^ absorption (Suksridechacin et al., 2020). Low dietary Mg^2+^ intake induced intestinal Mg^2+^ absorption (de Baaj et al., 2015; Groenestege et al., 2006) and subsequently increased circulating FGF‐23 levels (Matsuzaki et al., 2016). Although FGF‐23 diminished 1,25(OH)_2_D_3_‐induced intestinal Ca^2+^ absorption (Khuituan et al., 2012), its action on Mg^2+^ absorption is still unknown. Therefore, we aimed to elucidate the systemic and direct effects of PTH and FGF‐23 on small intestinal Mg^2+^ absorption.

## 2 METHODS

### 2.1 Animals

Male Sprague‐Dawley rats were obtained from the Nomura Siam International Co. Ltd. (Bangkok, Thailand). They were acclimatized for 7 days and maintained in open‐top plastic cages at 20 C–25 C and a 12‐h light‐dark cycle. The rats were fed with standard chow (Nomura Siam International Co. Ltd.) and reverse osmosis water ad libitum. Bodyweight, food intake, and water intake were recorded daily. All experiments were performed following relevant guidelines and regulations, including the ARRIVE guidelines (http://www.ARRIVEguidelines.org), and approved by the Ethics Committee on Animal Experiments, Burapha University, Thailand (IACUC 017/2562).

### 2.2 Experimental design

**FIGURE 1:**
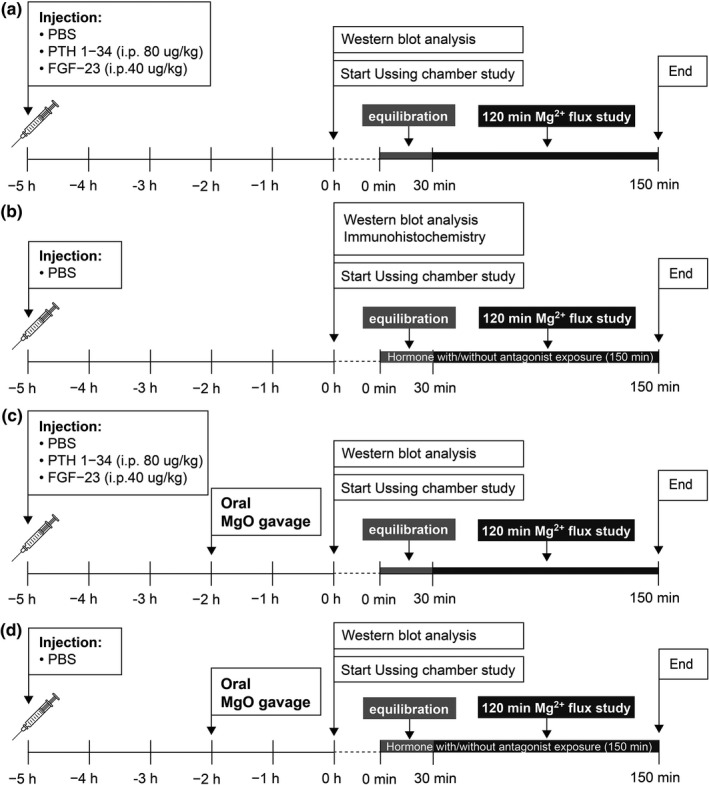
Representative diagrams show the experimental design (a–d)

To study the systemic effect of PTH and FGF‐23 on intestinal Mg^2+^ absorption (Figure 1a), the rats were intraperitoneal injected with phosphate‐buffered saline (PBS), 80 µg/kg PTH 1–34 (Sigma, St. Louis, MO, United States), or 40 µg/kg recombinant FGF‐23 (MyBioSource, San Diego, California, USA). The dose of PTH and FGF‐23 were comparable to that of previous studies (Brown et al., 2018; Chen et al., 2003; Faul et al., 2011). After 5 h, the rats were anesthetized with 70 mg/kg thiopental (Anesthal, Jagsonpal Pharmaceuticals Ltd., India) and blood was collected from the left ventricle followed by sacrifice. The duodenum, jejunum, and ileum were removed for western blot and Mg^2+^ transport studies.

To investigate the direct effects of PTH and FGF‐23 on small intestinal Mg^2+^ absorption, the duodenum, jejunum, and ileum of vehicle‐injected rats, which had been removed under anesthesia, were subjected to western blot, immunohistochemical, and Mg^2+^ flux analyses. Western blot and immunohistochemical analyses were done to observe the expression of FGF receptor 1 (FGFR1), PTH receptor 1 (PTHR1), α‐Klotho, TRPM6, and CNNM4 (Figure 1b). In the Ussing chamber setups, samples were treated with 1 ng/ml PTH 1–34, 1 ng/ml heat‐inactivated PTH 1–34, 20 ng/ml FGF‐23, 20 ng/ml heat‐inactivated FGF‐23, 20 ng/ml FGF‐23 plus 1 ng/ml PTH 1–34, 1 ng/ml PTH 1–34 with 1 µmol/l PTHrP 7–34 (PTHR antagonist; PromoCell GmbH, Heidelberg, Germany), or 20 ng/ml FGF‐23 with 1 µmol/l PD173074 (FGFR antagonist; Abcam, Cambridge, UK) for 150 min for the Mg^2+^ transport study. The concentrations of direct FGF‐23 and PTH 1–34 incubations were comparable to high physiological serum concentrations which were previously reported (Chen et al., 2021; Urakawa et al., 2006. The concentrations of antagonists were reported previously (Khuituan et al., 2012; Laohapitakworn et al., 2011; Maycas et al., 2015). During the Ussing chamber setup, the intestinal tissues were also incubated for 150 min with hormone plus a protein kinase A (PKA) antagonist (30 µmol/l H89, Calbiochem, San Diego, CA, USA), protein kinase C (PKC) antagonist (1 µmol/l Gö 6850, Sigma), mitogen‐activated protein kinases (MAPK) antagonist (1 µmol/l U‐0126, Calbiochem), or phosphoinositide 3‐kinase (PI3K, 75 µmol/l LY‐294002, Calbiochem).

To determine whether systemic PTH and FGF‐23 treatment modulates *in vivo* intestinal Mg^2+^ absorption (Figure 1c), the rats which were injected with hormone for 3 h subsequently received distilled water, 0.5 g/kg, or 1 g/kg MgO gavage. The volume of oral gavage was 5 ml/kg. After 2 h of oral gavage, blood was collected from the left ventricle under anesthesia and before the rats were sacrificed. The oral gave was performed as previously described (Dhande et al., 2009; Kh et al., 2000; Ozturk et al., 2016). The duodenum, jejunum, and ileum were collected immediately and subjected to western blot and Mg^2+^ transport studies. In another set of experiments, the rats were injected with hormone for 3 h before receiving oral Mg^2+^ supplement and subsequently housed for an additional 24 h in a metabolic cage to collect urine. The concentration of Mg^2+^ in serum and urine was measured by Labhouse Chonburi Co. Ltd. (Chonburi, Thailand).

The vehicle‐injected rats received oral Mg^2+^‐containing or Mg^2+^‐free supplement 2 h before performing the Mg^2+^ flux study (Figure 1d). The duodenum, jejunum, and ileum were exposed for 150 min during the Mg^2+^ flux study with 1 ng/ml PTH 1–34 or 20 ng/ml FGF‐23. The expression of TRPM6 and CNNM4 were determined by western blot analysis.

### 2.3 Magnesium flux measurement

The rate of the total, paracellular, and transcellular Mg^2+^ transport of duodenum, jejunum, and ileum was performed as previously described (Suksridechacin et al., 2020). In brief, the duodenum, jejunum, and ileum from each rat were dissected into two pieces, which then were mounted onto individually modified Ussing chamber setups. They were bathed and equilibrated for 30 min with a physiological bathing solution containing (in mmol/ml) 118 NaCl, 4.7 KCl, 1.1 MgCl_2_, 1.25 CaCl_2_, 23 NaHCO_3_, 12 D‐glucose, 2.5 L‐glutamine, and 2 D‐mannitol (osmolarity of 290–295 mmol/kg H_2_O and pH of 7.4). The solutions in Ussing chamber setup were maintained at 37 °C and continuously gassed with 5% CO_2_ in 95% O_2_. To study the rate of total Mg^2+^ transport in the first sample of each segment, the apical solution was 40 mmol/l MgCl_2_‐containing bating solution ([in mmol/L] 40 MgCl_2_, 2.5 CaCl_2_, 4.5 KCl, 12 D‐glucose, 2.5 L‐glutamine, 115 mannitol, and 10 HEPES pH 7.4), meanwhile, the basolateral solution was MgCl_2_‐free bating solution ([in mmol/L] 1.25 CaCl_2_, 4.5 KCl, 12 D‐glucose, 2.5 L‐glutamine, 250 D‐mannitol, and 10 HEPES pH 7.4). To study the rate of paracellular Mg^2+^ transport, another piece of each intestinal segment was incubated with apical 40 mmol/l MgCl_2_‐containing bating solution plus potent TRPM6 and Mg^2+^ channels blocker Co(III)hexamine (Wolf et al., 2010) (1 mmol/L; Sigma) and basolateral MgCl_2_‐free bating solution. Inhibition of TRPM6 and Mg^2+^ channels abolished transcellular Mg^2+^ transport. After 30, 60, and 120 min, a 100 µL solution was collected from the basolateral and apical sides. The Mg^2+^ concentration and the rate of Mg^2+^ transport were determined as previously described (Suksridechacin et al., 2020). The rate of transcellular Mg^2+^ transport in each intestinal segment was calculated by subtracting the rate of total Mg^2+^ transport with the rate of paracellular Mg^2+^ transport from the same intestinal segment of the individual rats (Suksridechacin et al., 2020).

### 2.4 Western blot analysis

Western blot analysis was performed following our previous method (Suksridechacin et al., 2020). The duodenum, jejunum, and ileum cells were collected by scraping the mucosal surface with an ice‐cold glass slide and lysed in cold Piece^®^ Ripa Buffer (Thermo Fisher Scientific Inc., Rockford, IL, USA) with 10% v/v protease inhibitor cocktail (Sigma) before sonicated and centrifuged at 12,000 g for 15 min. Membranous and cytosolic proteins were collected by using Mem‐PER™ Plus Membrane Protein Extraction Kit (Thermo Fisher Scientific Inc.). The membrane was probed with 1:1000 primary antibodies raised against CNNM4 (catalog no. SC‐68437; Santa Cruz Biotechnology, Santa Cruz, CA, USA), TRPM6 (catalog no. PA5‐77326; Thermo Fisher Scientific Inc.), PTHR1 (catalog no. MBS9706527; MyBioSource), FGFR1 (catalog no. ab10646; Abcam, Cambridge, UK), Klotho (catalog no. PA5‐21078; Thermo Fisher Scientific Inc), or β‐actin (catalog no. ab8226; Abcam). The membrane was subsequently incubated with 1:5000 HRP‐conjugated secondary antibodies (catalog no. ab97110 or ab6721; Abcam, catalog no. AP124P or AP136P; EMD Millipore), visualized by Thermo Scientific SuperSignal^®^ West Pico Substrate (Thermo Fisher Scientific Inc.), and captured by the ChemiDoc™ Touch Imaging System (Bio‐Rad, Hercules, CA, USA). Densitometric analysis was performed using ImageJ for Mac Os X (Schneider et al., 2012).

### 2.5 Statistical analysis

Results were expressed as means ± SE. Two sets of data were compared using the unpaired Student's *t*‐test. One‐way analysis of variance (ANOVA) with Dunnett's posttest was used for the comparison of multiple sets of data. All data were analyzed by GraphPad Prism for Mac Os (GraphPad Software Inc., San Diego, CA, USA).

## 3 RESULTS

### 3.1 Small intestinal epithelial cells express FGFR and PTHR proteins

**FIGURE 2:**
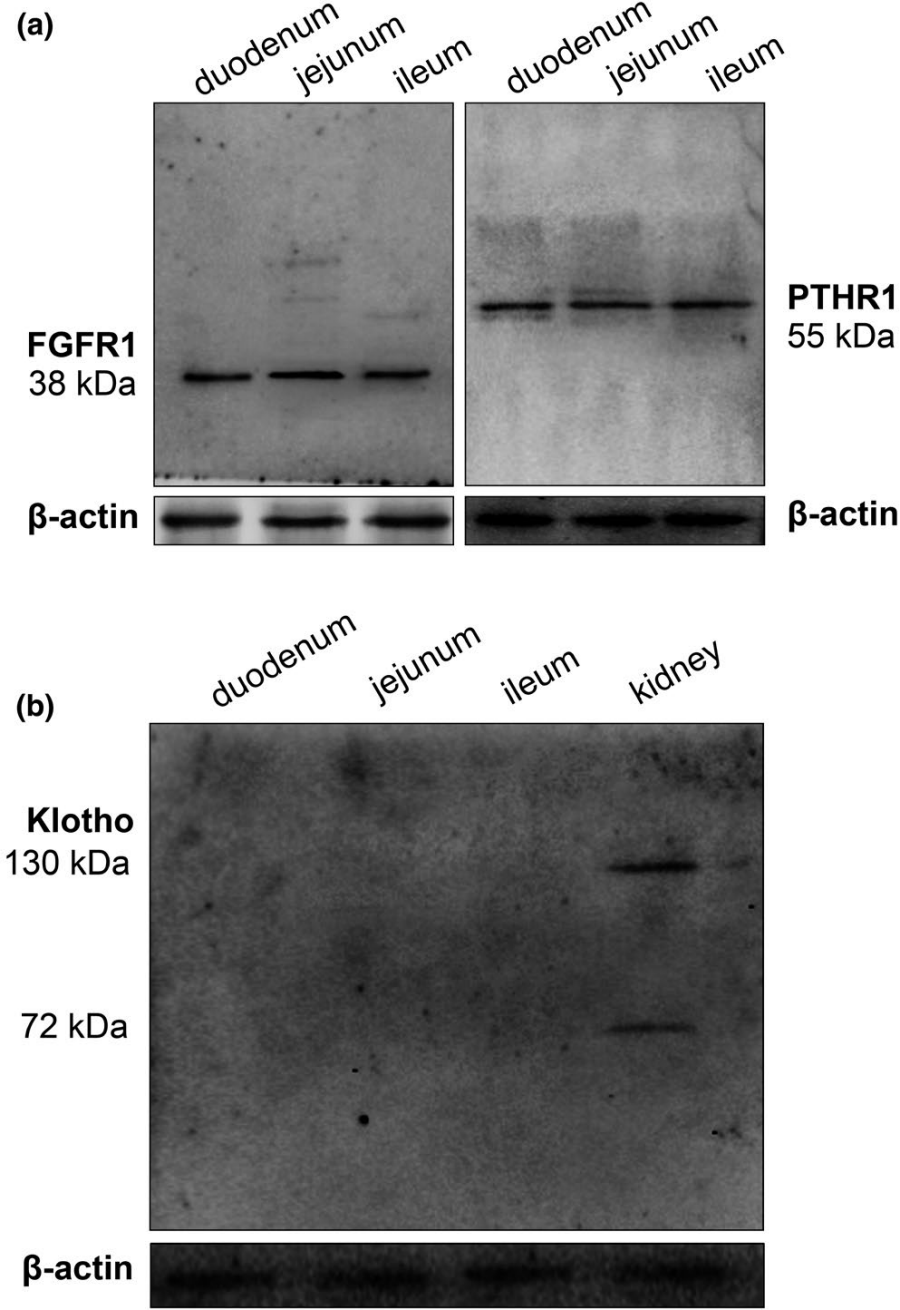
The FGFR1 and PTHR1 protein expression in duodenum, jejunum, and ileum of Sprague‐Dawley rats (a). The Klotho protein expression (b) in duodenum, jejunum, ileum, and kidney of Sprague‐Dawley rats

The expression of FGFR and PTHR was previously shown in the murine small intestine (Gentili et al., 2003; Khuituan et al., 2012). Western blot analysis showed that the duodenum, jejunum, and ileum abundantly expressed FGFR and PTHR proteins (Figure 2a). The expression of FGFR and PTHR suggested that the duodenum, jejunum, and ileum could directly respond to FGF‐23 and PTH exposure. However, Klotho protein was not expressed in the duodenum, jejunum, or ileum (Figure 2b). Consistent with a previous report (Khuituan et al., 2012), α‐ and β‐Klotho proteins were not expressed in the murine small intestine. Thus, FGF‐23 regulated murine small intestinal function possibly through a Klotho‐independent or circulating Klotho‐dependent mechanism (Khuituan et al., 2012).

### 3.2 FGF‐23 and PTH suppress small intestinal Mg^2+^ absorption

**FIGURE 3:**
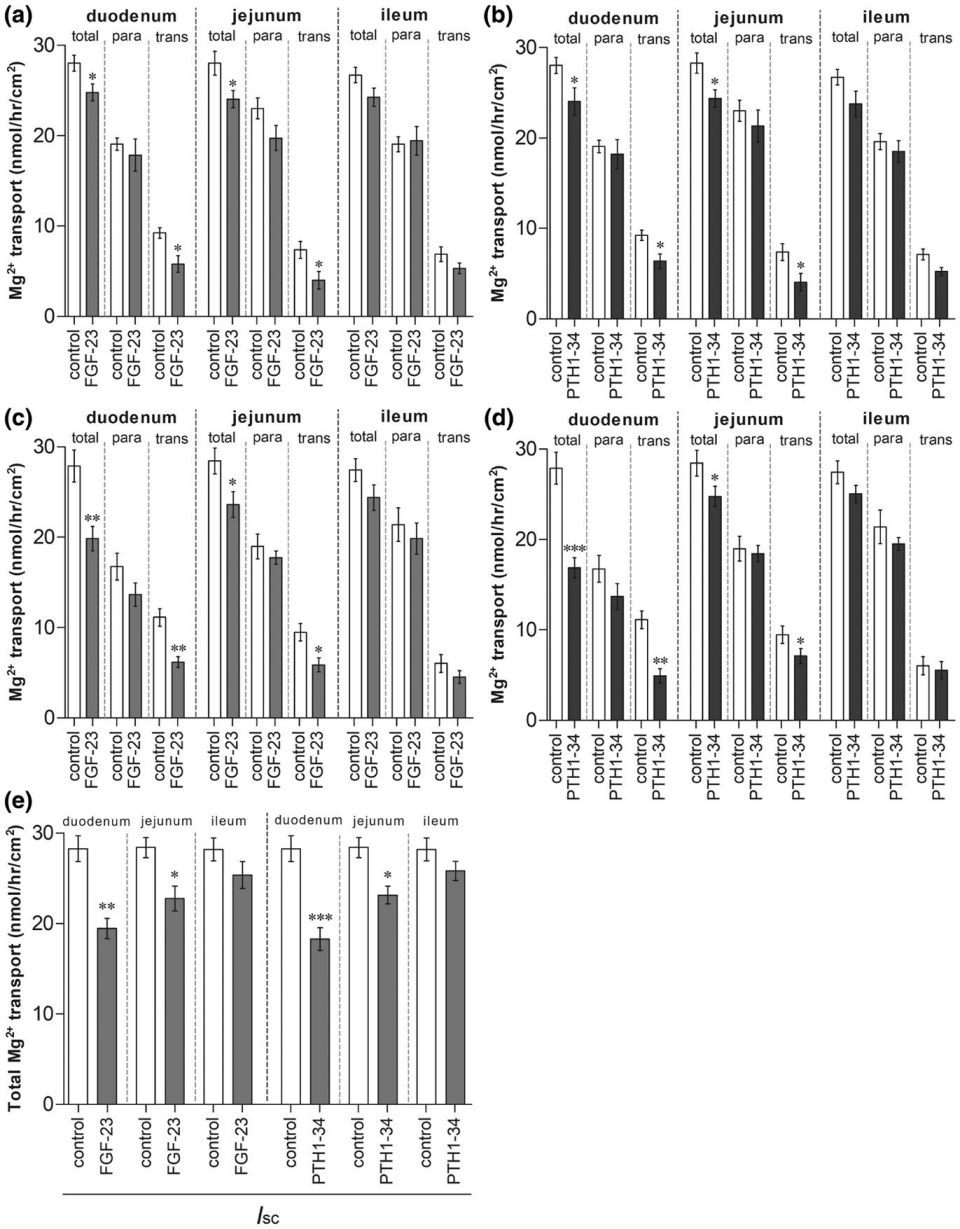
The systemic effect of FGF‐23 injection (a) or PTH injection (b) on Mg^2+^ transport across duodenum, jejunum, and ileum. The direct effect of direct FGF‐23 (c) or PTH (d) exposure on Mg^2+^ transport across duodenum, jejunum, and ileum. The rate of Mg^2+^ transport in duodenum, jejunum, and ileum tissues which continuously received *I*_sc_ (e). total; total Mg^2+^ transport, para; paracellular Mg^2+^ transport, trans; transcellular Mg^2+^ transport. ^*^ *p* < 0.05 compared with the corresponding control group. (*n* = 6)

As shown in Figure 3a, FGF‐23 injection for 5 h significantly suppressed the rate of total and transcellular Mg^2+^ transport in the duodenum and jejunum, but not in the ileum. PTH 1–34 injection also suppressed the rate of total and transcellular Mg^2+^ transport in the duodenum and jejunum (Figure 3b). Direct FGF‐23 exposure in the Ussing chamber significantly inhibited the rate of total and transcellular Mg^2+^ transport in the duodenum and jejunum, but not in the ileum (Figure 3c). PTH 1–34 also directly suppressed the rate of total and transcellular Mg^2+^ transport in the duodenum and jejunum (Figure 3d). Total Mg^2+^ transport represents the combination of transcellular and paracellular Mg^2+^ transport; therefore, acute systemic and direct FGF‐23 and PTH exposure suppressed transcellular Mg^2+^ absorption in the duodenum and jejunum of the rats.

We also measured transepithelial electrical parameters in the duodenum, jejunum, and ileum after 150 min of Mg^2+^ transport study (Table 1). The presence of electrical parameters confirmed that all intestinal tissues were still alive throughout the experimental period in Ussing chambers. FGF‐23 and PTH 1–34 significantly suppressed transepithelial potential differences (PD) and short‐circuit currents (*I*sc) in the duodenum and jejunum. Whereas transepithelial electrical resistant (TER) was significantly increased in the FGF‐23‐ or PTH 1–34‐exposed duodenum and jejunum. The increment of TER and decrement of *I*sc was consistent with the suppression of Mg^2+^ transport in the duodenum and jejunum of FGF‐23‐ or PTH 1–34‐exposed rats. However, the change in PD probably affected transepithelial Mg^2+^ transport. Therefore, we performed another set of Mg^2+^ transport experiments. The small intestinal tissues continuously received *I*_sc_ to nullify PD and to abolish voltage‐dependent Mg^2+^ transport. As demonstrated in Figure 2e, *I*_sc_ had no effect on the rate of total Mg^2+^ transport in the control or hormone–exposed duodenum, jejunum, and ileum. These results suggest that small intestinal Mg^2+^ transport occurred by a trans‐epithelium voltage‐independent mechanism.

**TABLE 1:** Transepithelial electrical parameters for duodenum, jejunum, and ileum

Group	Duodenum			Jejunum			Ileum		
	PD (mV)	*I*sc (μA/cm^2^)	TER (Ω/cm^2^)	PD (mV)	*I*sc (μA/cm^2^)	TER (Ω/cm^2^)	PD (mV)	*I*sc (μA/cm^2^)	TER (Ω/cm^2^)
Vehicle	4.13 ± 0.12	31.67 ± 1.76	131.16 ± 6.88	3.16 ± 0.21	20.60 ± 1.21	153.31 ± 4.69	3.58 ± 0.20	39.50 ± 1.94	90.59 ± 3.49
FGF−23	2.43 ± 0.15***	13.33 ± 1.20***	183.81 ± 8.46***	2.85 ± 0.13	15.25 ± 0.85**	187.42 ± 5.49**	3.48 ± 0.14	42.60 ± 1.91	81.97 ± 2.95
PTH1‐34	2.77 ± 0.20**	17.33 ± 1.86***	163.33 ± 6.01*	2.80 ± 0.21	15.75 ± 0.63*	177.28 ± 8.80*	3.93 ± 0.20	44.67 ± 1.76	87.98 ± 1.16
0.5 g/kg Mg^2+^ supplement									
Vehicle	4.17 ± 0.15	32.33 ± 1.86	129.53 ± 7.30	3.30 ± 0.23	20.80 ± 0.86	158.07 ± 6.84	3.53 ± 0.23	38.75 ± 1.49	91.07 ± 5.32
FGF−23	2.40 ± 0.06***	12.67 ± 0.33***	189.96 ± 9.45**	2.88 ± 0.06	15.25 ± 0.85*	190.29 ± 11.46*	3.38 ± 0.15	42.80 ± 1.83	79.29 ± 3.70
PTH1‐34	2.83 ± 0.15***	15.50 ± 0.66***	182.22 ± 5.43**	2.73 ± 0.13	14.25 ± 0.48*	191.31 ± 7.08*	3.98 ± 1.10	44.50 ± 1.20	89.14 ± 5.45
1 g/kg Mg^2+^ supplement									
Vehicle	3.35 ± 0.14	29.25 ± 1.11	115.30 ± 8.06	2.70 ± 0.20	26.25 ± 0.75	103.46 ± 9.44	4.03 ± 0.17	43.50 ± 1.04	92.86 ± 5.53
FGF−23	2.30 ± 0.19**	36.00 ± 2.74	64.68 ± 5.82**	2.55 ± 0.17	33.25 ± 1.65**	76.58 ± 2.39*	3.90 ± 0.11	41.50 ± 1.71	94.22 ± 2.57
PTH1‐34	2.74 ± 0.20	33.80 ± 2.20	83.27 ± 10.23*	2.95 ± 0.06	36.25 ± 0.48**	81.37 ± 1.32*	3.95 ± 0.21	40.50 ± 1.55	97.78 ± 5.36

Note

Values are means ± SE for transepithelial potential difference (PD), short‐circuit current (*I*sc), and transepithelial resistance (TER) in duodenum, jejunum, and ileum.

*p < 0.05, **p < 0.01, ***p < 0.001 compared with the vechicle‐injected control group.

### 3.3 Inhibitory effect of FGF‐23 and PTH is mediated by PKC

**FIGURE 4:**
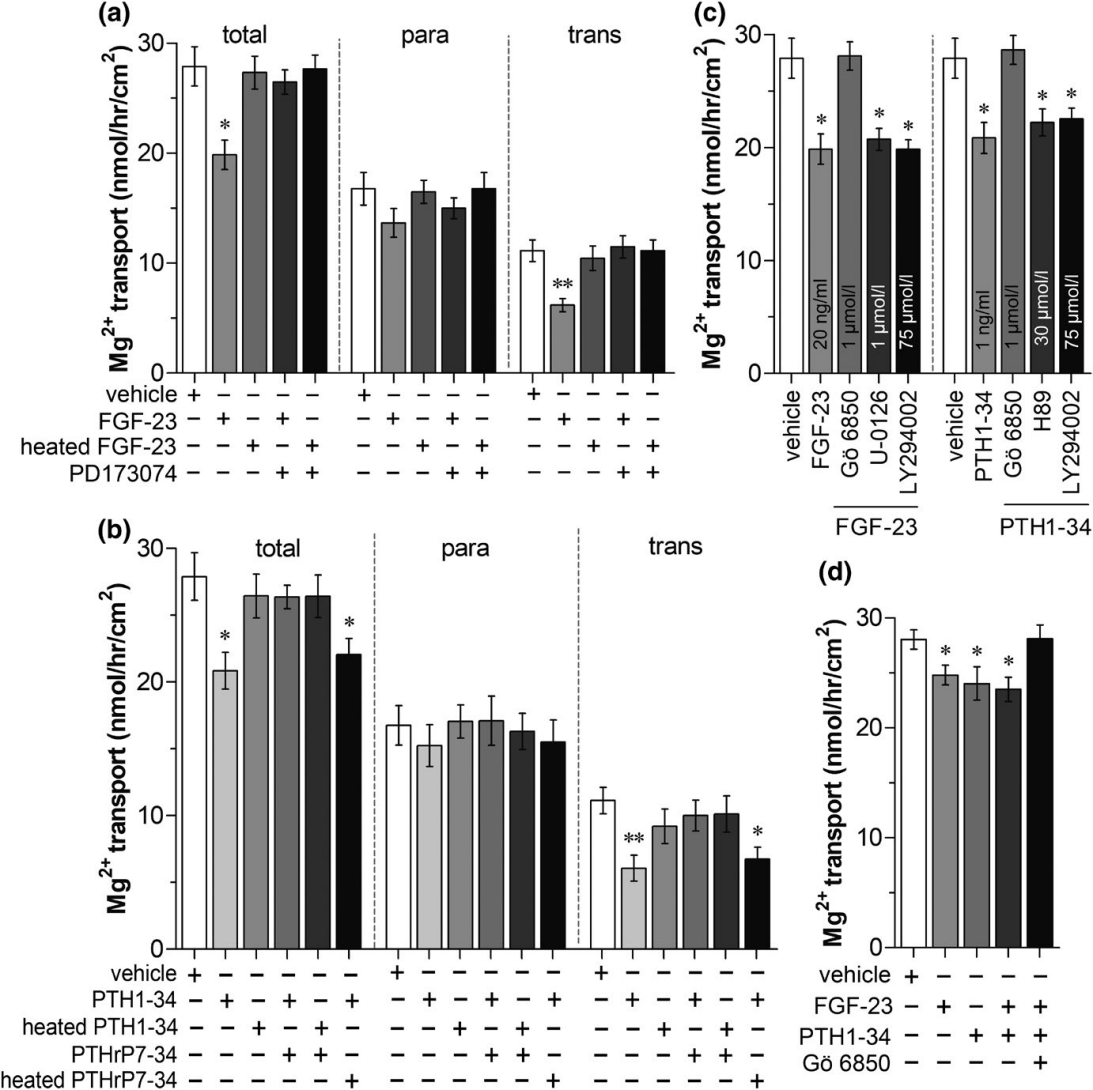
Duodenal Mg^2+^ transport in direct FGF‐23‐exposure with or without FGFR antagonist (a), direct PTH‐exposure with or without PTHR antagonist (b), direct FGF‐23‐ or direct PTH‐exposure with or without signaling inhibitors (c), and direct FGF‐23‐, direct PTH‐, or direct FGF‐23 plus PTH‐exposure with or without signaling inhibitor (d). total; total Mg^2+^ transport, para; paracellular Mg^2+^ transport, trans; transcellular Mg^2+^ transport. ^*^ *p* < 0.05, ^**^ *p* < 0.01 compared with the corresponding control group. (*n* = 6)

As demonstrated in Figure 4a, the FGFR antagonist, PD173074, markedly abolished the inhibitory effect of FGF‐23 on total duodenal Mg^2+^ absorption. The active PTHR antagonist, PTHrR 7–34, also abolished the inhibitory action of active PTH 1–34 on duodenal Mg^2+^ absorption (Figure 4b). FGF‐23 modulated epithelial electrolyte transport through the MAPK, PKC, and PI3K signaling pathways (Khuituan et al., 2012;). Duodenal tissues were exposed to FGF‐23 with Gö 6850, U‐0126, or LY‐294002. We found that the PKC inhibitor, Gö 6850, significantly abolished the inhibitory effect of FGF‐23 on intestinal Mg^2+^ absorption (Figure 4c). In epithelial tissues, PTH exerted its action through the PKA, PKC, and PI3K signaling pathways (Laohapitakworn et al., 2011). In the present study, duodenal tissues were exposed to active PTH 1–34 plus H89, Gö 6850, or LY‐294002 for 150 min in Mg^2+^ flux experiments. As shown in Figure 4c, the PKC inhibitor, Gö 6850, significantly decreased the inhibitory effect of PTH 1–34 on duodenal Mg^2+^ absorption. As shown in Figure 4d, the rate of Mg^2+^ transport in the FGF‐23 plus PTH‐treated group was comparable to that of the FGF‐23‐ or PTH‐treated groups. The PKC inhibitor also abolished the inhibitory effect of FGF‐23 plus PTH 1–34 on intestinal Mg^2+^ absorption (Figure 4d). These results suggest that both FGF‐23 and PTH regulate small intestinal Mg^2+^ absorption through corresponding receptor‐PKC‐dependent mechanisms.

### 3.4 Oral Mg^2+^ supplement modulates intestinal Mg^2+^ absorption in FGF‐23 and PTH‐injected rats

**FIGURE 5:**
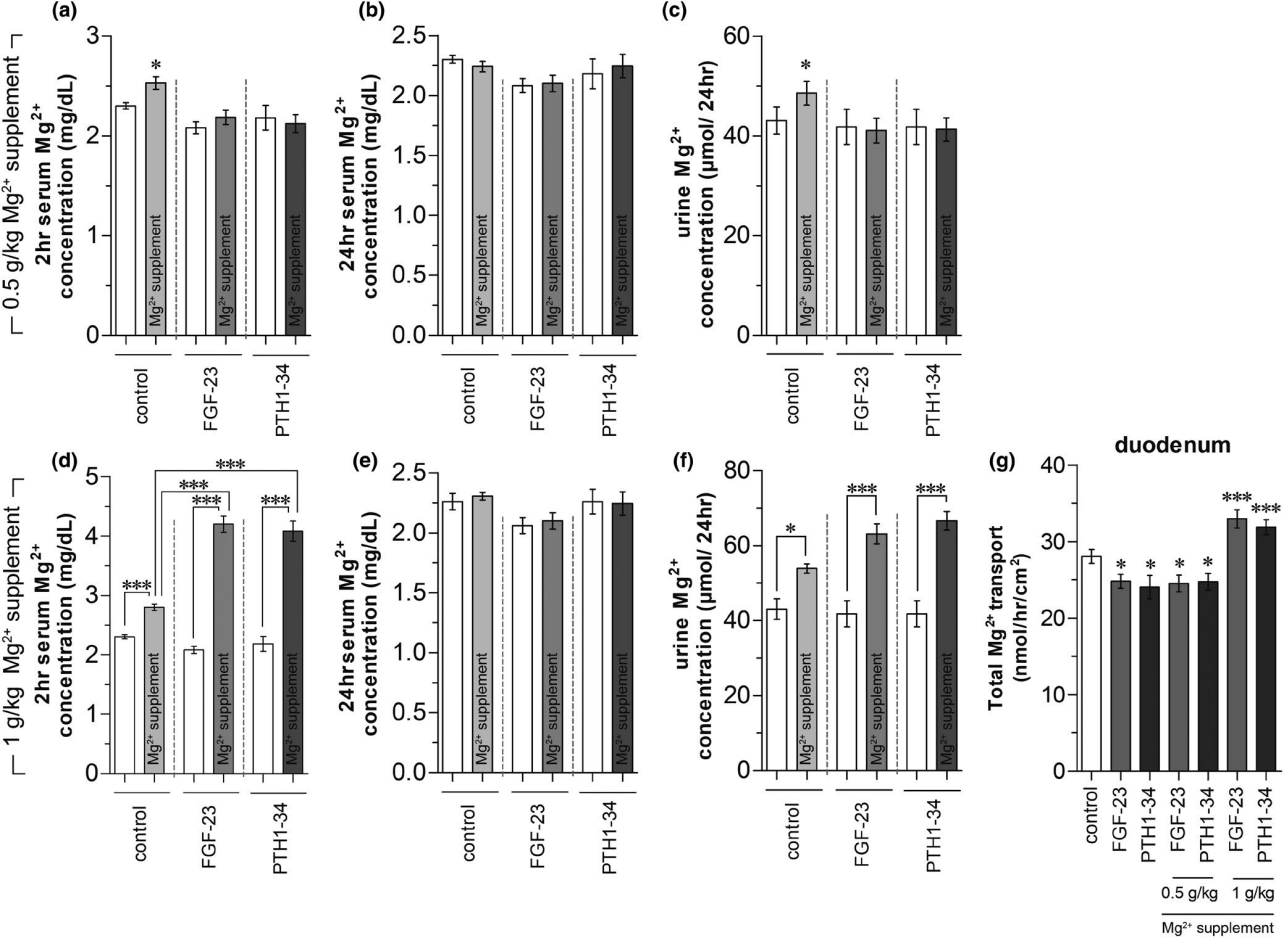
Effect of systemic FGF‐23‐ or PTH‐injection with or without 0.5 g/kg oral Mg^2+^ supplementation on 2 h plasma Mg^2+^ (a), 24 h plasma Mg^2+^ (b), and 24 h urine Mg^2+^ concentrations (c). Effect of systemic FGF‐23‐ or PTH‐injection with or without 1 g/kg oral Mg^2+^ supplementation on 2 h plasma Mg^2+^ (d), 24 h plasma Mg^2+^ (e), and 24 h urine Mg^2+^ concentrations (f). Effect of systemic FGF‐23‐ or PTH‐injection with 0.5 or 1 g/kg oral Mg^2+^ supplementation on duodenal Mg^2+^ absorption (g). ^*^ *p* < 0.05, ^**^ *p* < 0.01 compared with the corresponding control group. (*n* = 6)

**FIGURE 6:**
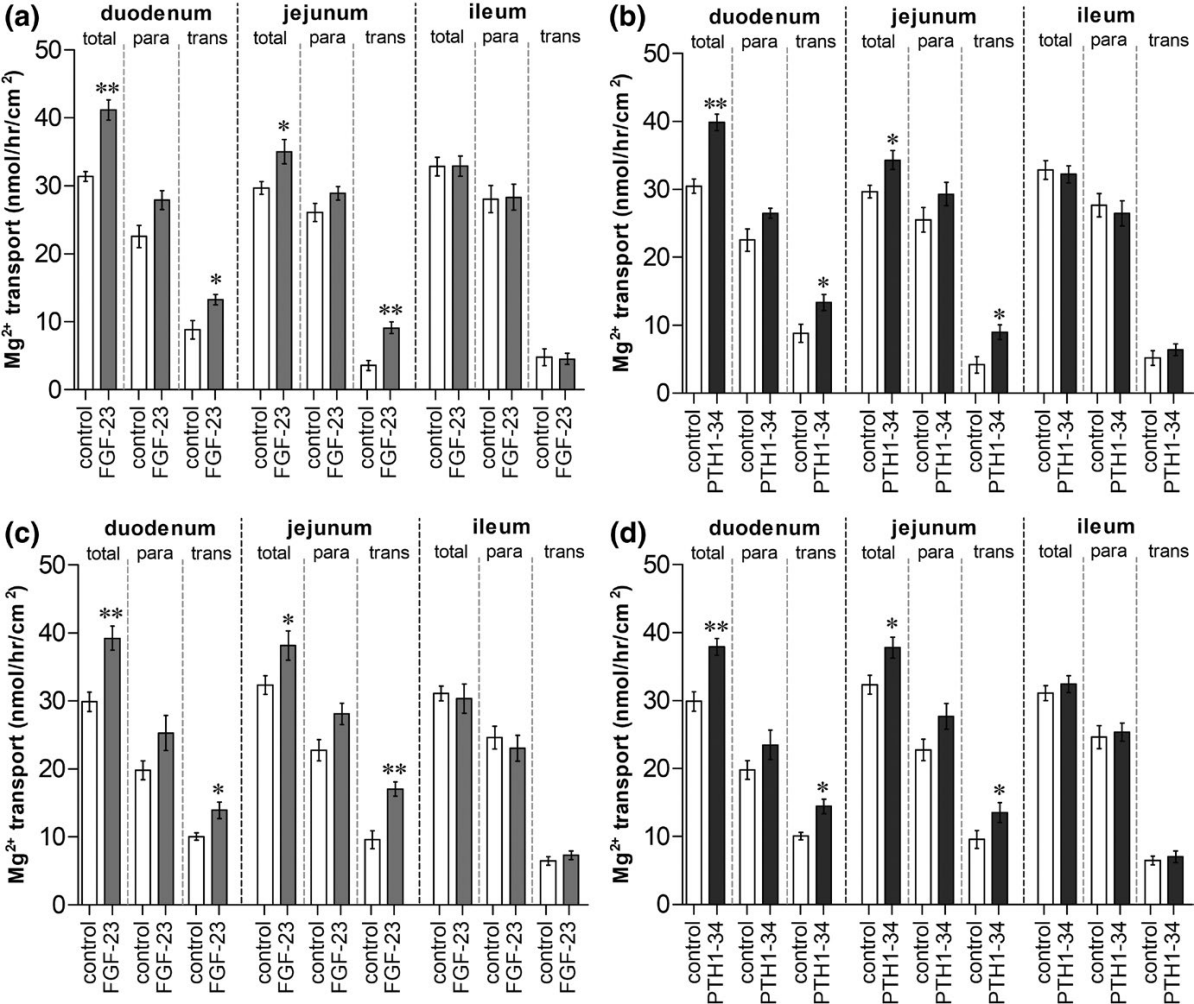
Effect of systemic FGF‐23‐ (a) or PTH‐injection (b) with 1 g/kg oral Mg^2+^ supplementation on duodenal, jejunal, and ileum Mg^2+^ absorption. The effect of direct FGF‐23 (c) or PTH exposure (d) on duodenal, jejunal, and ileum Mg^2+^ absorption of 1 g/kg oral Mg^2+^ supplemented rats. total; total Mg^2+^ transport, para; paracellular Mg^2+^ transport, trans; transcellular Mg^2+^ transport. ^*^ *p* < 0.05, ^**^ *p* < 0.01 compared with the corresponding control group. (*n* = 6)

To confirm the inhibitory effect of FGF‐23 and PTH on *in vivo* intestinal Mg^2+^ absorptions, after 3 h of hormone injection, the rats received 0.5 or 1 g/kg oral Mg^2+^ supplementation and subsequently measured plasma Mg^2+^ levels at 2 and 24 h after gavage. In vehicle‐injected rats, after 2 h of 0.5 g/kg oral Mg^2+^ supplementation plasma Mg^2+^ level significantly increased when compared with control rats (Figure 5a), indicating *in vivo* intestinal absorption after oral Mg^2+^ supplementation. After 24 h of gavage, the plasma Mg^2+^ level returned to normal (Figure 5b) with a higher urinary Mg^2+^ excretion (Figure 5c). In FGF‐23‐ or PTH 1–34‐injected rats, 0.5 g/kg oral Mg^2+^ supplementation did not affect plasma or urine Mg^2+^ levels (Figures 5, 6). These results confirmed that acute systemic FGF‐23 and PTH injection suppressed *in vivo* intestinal Mg^2+^ absorption.

As demonstrated in Figure 5d, after 2 h of 1 g/kg oral Mg^2+^ supplementation, plasma Mg^2+^ levels in the control rats significantly increased compared with non‐gavaged rats. Unexpectedly, in FGF‐23‐ or PTH 1–34‐injected rats, Mg^2+^ supplementation markedly increased plasma Mg^2+^ levels compared with non‐gavaged rats (Figure 5d). Among Mg^2+^‐supplemented rats, FGF‐23‐ or PTH 1–34‐exposure significantly increased plasma Mg^2+^ concentration compared with the vehicle‐injected rats (Figure 5d). After 24 h of 1 g/kg Mg^2+^ supplementation, plasma Mg^2+^ levels in the rats returned to a normal level (Figure 5e) with higher urine Mg^2+^ excretion (Figure 5f). We further performed a duodenal Mg^2+^ transport study in the rats that received oral Mg^2+^ supplementation. As demonstrated in Figure 5g, 0.5 g/kg Mg^2+^ supplementation did not affect FGF‐23‐ or PTH 1–34‐suppressed duodenal Mg^2+^ absorption. In contrast, 1 g/kg oral Mg^2+^ supplementation significantly increased duodenal Mg^2+^ absorption in both FGF‐23‐ and PTH 1–34‐injected rats when compared with the vehicle‐injected rats. These results suggest that 1 g/kg Mg^2+^ supplementation stimulated intestinal Mg^2+^ absorption in PTH‐ and FGF‐23‐injected rats.

We further studied the effect of 1 g/kg oral Mg^2+^ supplementation on the modulating effect of FGF‐23 and PTH on duodenal, jejunal, and ileal Mg^2+^ absorption. Systemic FGF‐23 injection plus 1 g/kg Mg^2+^ supplementation significantly increased total and transcellular Mg^2+^ transport in the duodenum and jejunum (Figure 6a). PTH 1–34 injection with 1 g/kg Mg^2+^ supplementation also markedly increased total and transcellular Mg^2+^ transport in the rat duodenum and jejunum (Figure 6b). Direct FGF‐23 exposure in the Ussing chamber significantly enhanced total and transcellular Mg^2+^ transport in the duodenum and jejunum (Figure 6c) of 1 g/kg Mg^2+^ supplemented rats compared with control rats. Direct PTH 1–34 exposure also increased total and transcellular Mg^2+^ transport in the rat duodenum and jejunum of 1 g/kg Mg^2+^‐supplemented rats (Figure 6d). These results confirmed our previous results that high dose 1 g/kg oral Mg^2+^ gavage reversed the inhibitory effect of systemic and direct PTH‐ and FGF‐23‐exposure on small intestinal transcellular Mg^2+^ transport.

### 3.5 FGF‐23, PTH, and Mg^2+^ supplementation modulates intestinal TRPM6 and CNNM4 protein expression

**FIGURE 7:**
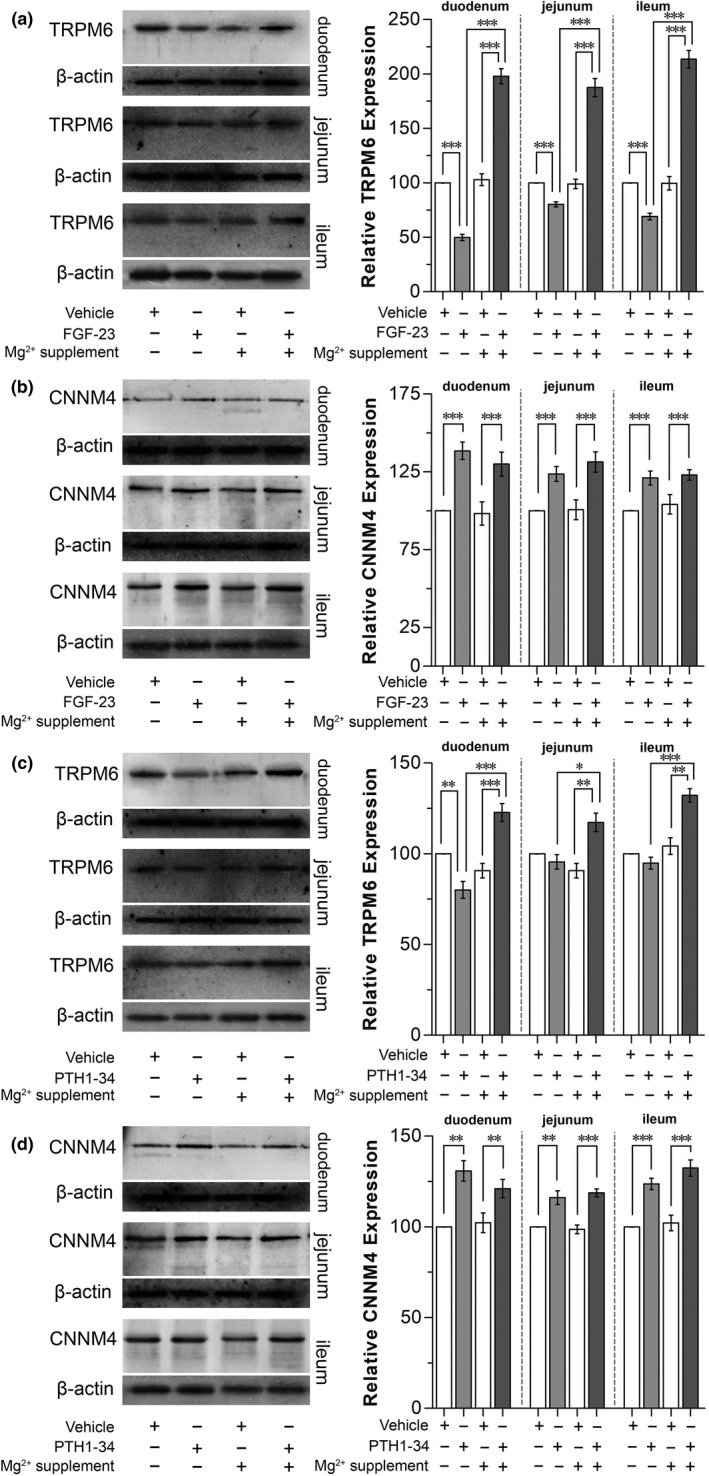
Total TRPM6 (a) or total CNNM4 (b) expression in duodenal, jejunum, and ileum of the rats that received systemic FGF‐23 injection with 1 g/kg oral Mg^2+^ supplementation. Total TRPM6 (c) or total CNNM4 (d) expression in duodenal, jejunum, and ileum of the rats that received systemic PTH‐23 injection with 1 g/kg oral Mg^2+^ supplementation. ^*^ *p* < 0.05, ^**^ *p* < 0.01, ^***^ *p* < 0.001. (*n* = 6)

Systemic FGF‐23 exposure significantly suppressed total TRPM6 (Figure 7a), but increased total CNNM4 protein expression (Figure 7b) in the duodenum, jejunum, and ileum. High dose 1 g/kg Mg^2+^ supplementation did not affect small intestinal total TRPM6 (Figure 7a) and CNNM4 protein expression (Figure 7b) compared with the control rats. High dose 1 g/kg Mg^2+^ supplementation plus systemic FGF‐23 injection significantly increased TRPM6 expression in the duodenum, jejunum, and ileum compared with Mg^2+^‐supplemented or FGF‐23‐injected rats (Figure 7a). Mg^2+^ supplementation did not affect FGF‐23‐induced CNNM4 expression (Figure 7b). Similar to FGF‐23, PTH 1–34 significantly decreased total TRPM6 expression (Figure 7c), but it increased total CNNM4 expression (Figure 7d) in rat small intestine. High dose Mg^2+^ supplementation also significantly induced TRPM6 expression in the small intestine of PTH 1–34‐injected rats compared with Mg^2+^ supplemented‐ or PTH 1–34‐injected rats (Figure 7c). Mg^2+^ supplementation did not affect PTH‐induced CNNM4 expression (Figure 7d).

**FIGURE 8:**
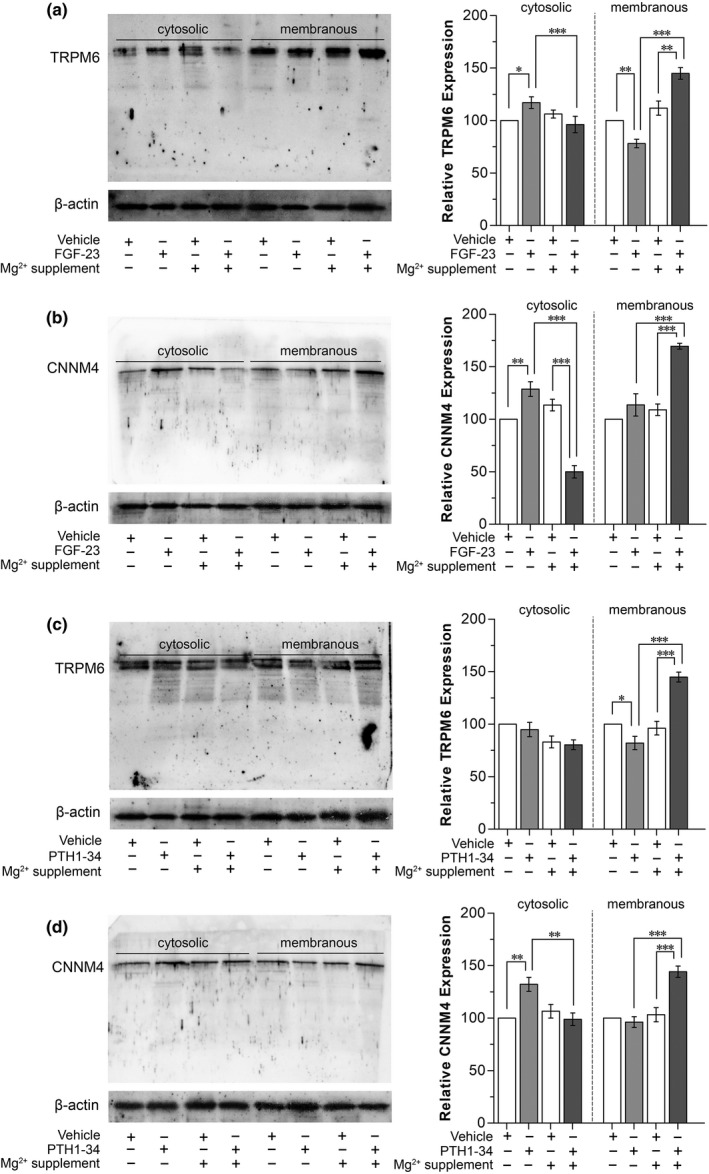
Duodenal cytosolic and membranous TRPM6 (a) or CNNM4 (b) expression of the rats that received systemic FGF‐23 injection with 1 g/k g/kg oral Mg^2+^ supplementation. Duodenal cytosolic and membranous TRPM6 (a) or CNNM4 (b) expression of the rats that received systemic PTH injection with 1 g/k g/kg oral Mg^2+^ supplementation. ^*^ *p* < 0.05, ^**^ *p* < 0.01, ^***^ *p* < 0.001. (*n* = 6)

Systemic FGF‐23 exposure decreased membranous, but increased cytosolic TRPM6 protein expression in the duodenum (Figure 8a). FGF‐23 injection plus 1 g/kg Mg^2+^ supplementation significantly increased membranous TRPM6 expression compared with Mg^2+^‐supplemented or FGF23‐injected rats (Figure 8a). FGF‐23 increased cytosolic CNNM4 protein expression (Figure 8b). FGF‐23 injection plus 1 g/kg oral Mg^2+^ supplementation significantly increased membranous CNNM4, but decreased cytosolic CNNM4 protein expression compared with Mg^2+^‐supplemented or FGF23‐injected rats (Figure 8b). In PTH 1–34‐injected rats, membranous TRPM6 was significantly decreased (Figure 8c). PTH 1–34 injection plus 1 g/kg oral Mg^2+^ supplementation significantly increased membranous TRPM6 (Figure 8c) and membranous CNNM4 (Figure 8d) expression compared with Mg^2+^‐supplemented or PTH 1–34‐injected rats.

Taken together, the results suggested that FGF‐23 and PTH suppressed small intestinal transcellular Mg^2+^ absorption, at least in part, by downregulating membranous TRPM6 expression. FGF‐23 or PTH plus 1 g/kg oral Mg^2+^ supplementation induced small intestinal membranous TRPM6 and CNNM4 expression which led to an increment in transcellular Mg^2+^ absorption in rat small intestine. This probably explains why 1 g/kg oral Mg^2+^ supplementation reverses the inhibitory effect of FGF‐23 and PTH on small intestinal Mg^2+^ absorption.

## 4 DISCUSSION

In the present study, we proposed a novel magnesiotropic action for PTH and FGF‐23 by suppressing small intestinal Mg^2+^ absorption, which is probably, implicated in the mechanism of PTH and FGF‐23 controlling plasma Ca^2+^ levels. During hypocalcemia, PTH solely triggers the bone resorption process, which increases plasma Ca^2+^, P_i_, and Mg^2+^ levels (Leaf & Christov, 2019; Zofkova & Kancheva, 1995). PTH activates renal Ca^2+^ and Mg^2+^ reabsorption, P_i_ excretion, and 1,25(OH)_2_D_3_ production. The 1,25–1,25(OH)_2_D_3_ induces intestinal Ca^2+^ absorption. The increment of plasma P_i_ and PTH activates FGF‐23 release which further abolishes 1,25(OH)_2_D_3_‐induced intestinal Ca^2+^ absorption and induces renal P_i_ excretion. PTH and FGF‐23 synergistically suppressed small intestinal absorption of dietary Mg^2+^. Therefore, PTH and FGF‐23 exert their harmonious effects in the regulation of plasma Ca^2+^ levels by preventing hyperphosphatemia and hypermagnesemia.

PTH and FGF‐23 increased TER which suppressed paracellular ion transport. However, PTH and FGF‐23 had no effect on paracellular Mg^2+^ transport. Transepithelial *I*sc indicates the charge flow across intestinal tissues over time. The decrement in *I*sc was consistent with low small intestinal Mg^2+^ absorption in FGF‐23‐ or PTH‐injected rats. We showed that voltage‐dependent transport was not involved in Mg^2+^ absorption; however, transepithelial ion transport produced PD. If present, the change in PD resulting from the change in Mg^2+^ transport (V_Δ_,_Mg_^2+^) may be calculated by the formula: V_Δ_,_Mg_^2+^ = *J*_Δ, Mg_^2+^ × z*F* × TER (*J*_Δ, Mg_^2+^ is the change in the rate of Mg^2+^ transport, z = +2, *F* = 96,485.34 C/mol). The rate of Mg^2+^ transport of the control, FGF‐23‐, and PTH‐exposed duodenum was 28.03, 24.79, and 24.03 nmol/hr/cm^2^, respectively. The TER of FGF‐23‐ and PTH‐exposed duodenum was 183.81 and 163.33 Ω⋅cm^2^, respectively. Hence, V_Δ_,_Mg_^2+^ in FGF‐23‐ and PTH‐exposed intestine was 31.92 and 35.02 µV, respectively, which were considered negligible.

In the present study, exogenous FGF‐23 or PTH injection inhibited *in vivo* intestinal Mg^2+^ absorptions in 0.5 g/kg oral Mg^2+^‐supplemented rats. In the Ussing chamber setups, systemic and direct exposure to FGF‐23 or PTH suppressed transcellular Mg^2+^ transport in the duodenum and ileum. The previous study reported that, among high dietary Mg^2+^ intake groups, the parathyroidectomized (PTX) rat showed significantly higher serum Mg^2+^ levels compared with that of the sham‐operated rats, whereas the urinary Mg^2+^ excretion of PTX and sham‐operated rats were comparable (Thumfart et al., 2008). This previous study suggested that endogenous PTH suppressed intestinal Mg^2+^ absorption. PTH directly induced intestinal HCO_3_^–^ secretion (Laohapitakworn et al., 2011) and antagonists of mucosal HCO_3_^–^ secretion induced duodenal Mg^2+^ absorption (Suksridechacin et al., 2020). Therefore, PTH may suppress intestinal Mg^2+^ absorption by a mucosal HCO_3_^–^ secretion‐dependent mechanism. There are no previous studies indicating a regulatory role of FGF‐23 on intestinal Mg^2+^ absorption. Previously, FGF‐23 abolished 1,25(OH)_2_D_3_‐induced transient receptor potential cation channel subfamily V (TRPV)‐5, TRPV‐6, and calbindin‐D9k expression, which suppressed duodenal Ca^2+^ absorption in 1,25(OH)_2_D_3_ pre‐treated rats (Khuituan et al., 2012). However, FGF‐23 had no direct inhibitory effect on duodenal Ca^2+^ absorption (Khuituan et al., 2012). The present study showed the systemic and direct inhibitory effect of FGF‐23 on intestinal Mg^2+^ absorption.

Regarding the synergistic inhibitory action of PTH and FGF‐23 on transcellular Mg^2+^ uptake in the duodenum and jejunum, they may suppress TRPM6 activity and/or expression. Both FGF‐23 and PTH exerted their inhibitory action through PKC activation. In renal tubular epithelium, FGF‐23 and PTH promoted P_i_ excretion through a sodium‐hydrogen exchanger regulatory factor‐1 (NHERF‐1)‐PKC‐dependent pathway by downregulating NaPi‐IIa and NaPi‐IIc expression (Weinman et al., 2011). In the rat small intestine, NHERF‐1 was highly expressed in the duodenum and jejunum and implicated in small intestinal P_i_ absorption (Giral et al., 2012). However, the role of NHERF‐1 on the synergistic inhibitory action of PTH and FGF‐23 on transcellular Mg^2+^ absorption in the duodenum and jejunum requires further study. Since NHERF‐1 is rarely expressed in rat ileum (Giral et al., 2012), this may explain why PTH and FGF‐23 did not affect ileal Mg^2+^ absorption. In human embryonic kidney (HEK) 293 cells, TRPM6 activity was inhibited by the repressor of estrogen receptor activity (REA) and receptor for activated C‐kinase 1 (RACK1) (Cao et al., 2008, 2009). The PKC agonist, PMA, potentiated the inhibitory action of REA and RACK1 on TRPM6 activity in renal‐derived HEK293 cells (Cao et al., 2008, 2009). These studies suggest that PKC activation probably increased transcellular Mg^2+^ reabsorption in renal tubular epithelium; whereas we propose that PKC activation suppresses small intestinal Mg^2+^ absorption. The contradictory effects of PKC activation on transepithelial Mg^2+^ transport between our study and that of others may have resulted from the use of different tissues. Moreover, the PKC activator, PMA, used in previous studies (Cao et al., 2008, 2009) may activate conventional PKC isoforms (α, β1, β2, and γ) and novel PKC isoforms (δ, ε, θ, and η) (Geraldes & King, 2010), whereas the PKC inhibitor, Gö 6850, used in the present study, may inhibit conventional (α, β1, β2, and γ) and novel PKC isoforms (δ and ε). Different PKC isoforms have dissimilar functions in the mucosal epithelium (Farhadi et al., 2006). The contradictory effects of PKC activation on the small intestine and renal cells probably resulted from the difference in PKC isoforms. Besides TRMP6 activity, FGF‐23 and PTH inhibited small intestinal Mg^2+^ absorption, at least in part, from the suppression of membranous and total TRPM6 protein expression by an unknown mechanism. In contrast, neither PTH nor PTX affected TRPM6 mRNA expression in the kidney (Groenestege et al., 2006). Therefore, PTH regulates TRPM6 expression in an organ‐specific manner.

We report for the first time that exogenous PTH and FGF‐23 injection upregulated CNNM4 protein expression in the rat small intestine with an unknown mechanism. Incremental CNNM4 expression is probably implicated in transcellular Mg^2+^ absorption or Ca^2+^‐dependent signaling events. In neurons, skeletal muscle, cardiac muscle, and pancreatic acini, intracellular Mg^2+^ plays a role as an antagonist of intracellular Ca^2+^‐dependent activities (de Baaij et al., 2015). For example, pancreatic exocrine action is a Ca^2+^‐calmodulin‐dependent mechanism that is potentiated by intracellular Mg^2+^. Postprandial acetylcholine and cholecystokinin 8 stimulated Mg^2+^ extrusion as well as intracellular Ca^2+^ release to stimulate pancreatic enzyme secretion (Singh and Wisdom, 1995; Wisdom et al., 1996). PTH and FGF‐23 induced CNNM4 expression perhaps to regulate enterocyte activities during intracellular Ca^2+^‐dependent mechanisms.

The high dose (1 g/kg) oral Mg^2+^ supplementation reversed the inhibitory effect of PTH and FGF‐23 on small intestinal Mg^2+^ absorption by upregulating membranous TRPM6 and membranous CNNM4 protein expression. In mice, 14 days of high dietary Mg^2+^ intake significantly increased colonic TRMP6 mRNA expression (Groenestege et al., 2006). In the present study, 2 h of 1 g/kg oral Mg^2+^ supplementation in hormonal‐injected rats markedly induced small intestinal membranous TRPM6 expression. High dose oral Mg^2+^ supplementation with systemic hormonal exposure also induced CNNM4 translocation from the cytosolic pool to the plasma membrane, as demonstrated by lower cytosolic CNNM4 and higher membranous CNNM4. However, a low dose (0.5 g/kg) of oral Mg^2+^ supplementation did not affect TRPM6 and CNNM4 expression (data not shown). The exact mechanism regarding the different effects of 0.5 and 1 g/kg oral Mg^2+^ supplementation on PTH‐ and FGF‐23‐modulated intestinal function could not be explained in the present study; however, the different effects of 0.5 and 1 g/kg Mg^2+^ supplementation were reported in a previous study (Dhande et al., 2009). Low dose (0.5 g/kg) oral Mg^2+^ supplementation significantly suppressed maximal electroshock seizures (MES) in rats, whereas 1 g/kg oral Mg^2+^ supplementation markedly enhanced phenytoin action in MES.

## 5 CONCLUSION

We proposed a novel Mg^2+^‐regulatory action for PTH and FGF‐23 by suppressing small intestinal Mg^2+^ absorption through a PKC‐dependent mechanism. PTH and FGF‐23 suppressed membranous TRPM6 protein expression, whereas it increased CNNM4 expression in the small intestine of the rats. High dose (1 g/kg) oral Mg^2+^ supplementation significantly reversed the inhibitory effect of PTH and FGF‐23 by upregulating membranous TRPM6 expression.

## CONFLICT OF INTEREST

The authors declare no competing interests.

## AUTHOR CONTRIBUTIONS

N.S. designed and performed experiments, analyzed interpreted the results, and wrote the manuscript; N.T. designed and performed experiments, analyzed and interpreted the results, and wrote and edited the manuscript. The authors declare that all data were generated in‐house and that no paper mill was used.
